# A chromosome-level genome assembly of the Chinese tupelo *Nyssa sinensis*

**DOI:** 10.1038/s41597-019-0296-y

**Published:** 2019-11-25

**Authors:** Xuchen Yang, Minghui Kang, Yanting Yang, Haifeng Xiong, Mingcheng Wang, Zhiyang Zhang, Zefu Wang, Haolin Wu, Tao Ma, Jianquan Liu, Zhenxiang Xi

**Affiliations:** 1 Key Laboratory of Bio-Resource and Eco-Environment of Ministry of Education, College of Life Sciences, Sichuan University, Chengdu, 610065 China; 2 State Key Laboratory of Grassland Agro-Ecosystems, College of Life Sciences, Lanzhou University, Lanzhou, 730000 China

**Keywords:** Genome, Plant sciences, Next-generation sequencing, DNA sequencing, RNA sequencing

## Abstract

The deciduous Chinese tupelo (*Nyssa sinensis* Oliv.) is a popular ornamental tree for the spectacular autumn leaf color. Here, using single-molecule sequencing and chromosome conformation capture data, we report a high-quality, chromosome-level genome assembly of *N*. *sinensis*. PacBio long reads were *de novo* assembled into 647 polished contigs with a total length of 1,001.42 megabases (Mb) and an N50 size of 3.62 Mb, which is in line with genome sizes estimated using flow cytometry and the *k*-mer analysis. These contigs were further clustered and ordered into 22 pseudo-chromosomes based on Hi-C data, matching the chromosome counts in *Nyssa* obtained from previous cytological studies. In addition, a total of 664.91 Mb of repetitive elements were identified and a total of 37,884 protein-coding genes were predicted in the genome of *N*. *sinensis*. All data were deposited in publicly available repositories, and should be a valuable resource for genomics, evolution, and conservation biology.

## Abstract (summary)

**Table:** 

Measurement(s)	whole genome sequencing assay • transcription profiling assay • sequence_assembly • sequence annotation
Technology Type(s)	DNA sequencing • RNA sequencing assay • genome assembly • bioinformatics analysis
Sample Characteristic - Organism	Nyssa sinensis
Sample Characteristic - Environment	terrestrial biome

Machine-accessible metadata file describing the reported data: 10.6084/m9.figshare.10069796

## Background & Summary

*Nyssa sinensis* Oliv., commonly known as Chinese tupelo, is a deciduous tree with ovate leaves, which turn brilliant red, orange, and yellow in autumn. It belongs to the family Nyssaceae within the order Cornales, and is native to southern China and Vietnam. The genus *Nyssa* comprises three species in North America (i.e., *N*. *aquatica*, *N*. *ogeche*, and *N*. *sylvatica*), three in eastern Asia (i.e., *N*. *javanica*, *N*. *sinensis*, and *N*. *yunnanensis*), and one in Costa Rica (i.e., *N*. *talamancana*)^1,2^. Thus, it is one of the plant genera that exhibit a classical disjunct distribution between eastern Asia and North America. In addition, the fossil record of *Nyssa* is very rich^1,3,4^, making it ideal for studying the evolutionary history of Tertiary relict floras.

The genome of *Camptotheca acuminata* (Nyssaceae) has recently been sequenced using the Illumina platform^5^, and the final assembly is 403.17 megabases (Mb) with a contig N50 size of 107.59 kilobases (kb). So far, this is the only sequenced genome within the order Cornales. Here, we utilized a combination of the PacBio long-read sequencing technology^6^ and the high-throughput chromosome conformation capture (Hi-C) technique^7^ to generate the genome sequence of *N*. *sinensis*. Long reads were *de novo* assembled into 647 polished contigs with a total length of 1,001.42 Mb and an N50 size of 3.62 Mb, which is in line with genome sizes estimated using flow cytometry and the *k*-mer analysis. These contigs were further clustered and ordered into 22 pseudo-chromosomes based on Hi-C data. Our results provide the first high-quality, chromosome-level genome assembly for the order Cornales, which should be a valuable resource for genomics, evolution, and conservation biology.

## Methods

### Sample collection and high-throughput sequencing

We sampled a single individual of *N*. *sinensis* from the Kunming Botanical Garden, Yunnan, China. The total genomic DNA was extracted from fresh leaves using a modified CTAB method^8^, and sequenced using the PacBio Sequel System (for genome assembly) and the Illumina HiSeq. 4000 System (for genome survey and base level correction after the assembly). Here, one library with an insertion size of 350 bp was prepared for the Illumina platform and 20-kb libraries were constructed for the PacBio platform according to the manufacturers’ protocols. A total of 104.34 gigabases (Gb) of polymerase reads were generated using the PacBio platform, and a total of 104.19 Gb (coverage of 99.12×) of subreads were obtained after removing adaptors in polymerase reads (Table 1). The N50 read length reached 22.26 kb and 14.53 kb for polymerase reads and subreads, respectively. A total of 58.92 Gb of 150-bp paired-end reads were generated using the Illumina platform, and a total of 58.83 Gb (coverage of 55.97×) of reads were obtained after adapter trimming and quality filtering (Table 1). In addition, the Hi-C library was constructed using young leaf tissue from the same individual of *N*. *sinensis*, and sequenced using the Illumina platform. A total of 126.81 Gb (coverage of 120.63×) of 150-bp paired-end reads were obtained after adapter trimming and quality filtering (Table 1), which were later applied to extend the contiguity of the genome assembly to the chromosomal level. Furthermore, leaves and flowers were collected from the same individual of *N*. *sinensis*, and RNA-Seq reads were generated for genome annotation using the Illumina platform. A total of 17.36 Gb of 150-bp paired-end reads were obtained after adapter trimming and quality filtering (Table 1).

**Table 1:** Summary of sequencing data generated in this study.

Library type	Platform	Read length	Clean reads	Clean base	Coverage	Application
Long reads	PacBio Sequel	14,526 bp (N50)	11,197,047	104.19 Gb	99.12×	Genome assembly
Short reads	HiSeq. 4000	2 × 150 bp	2 × 196,110,604	58.83 Gb	55.97×	Genome survey and base level correction
Hi-C	HiSeq. 4000	2 × 150 bp	2 × 423,362,084	126.81 Gb	120.63×	Chromosome construction
RNA-Seq	HiSeq. 4000	2 × 150 bp	2 × 57,866,710	17.36 Gb	—	Genome annotation

### Genome size and heterozygosity estimation

**Fig. 1:**
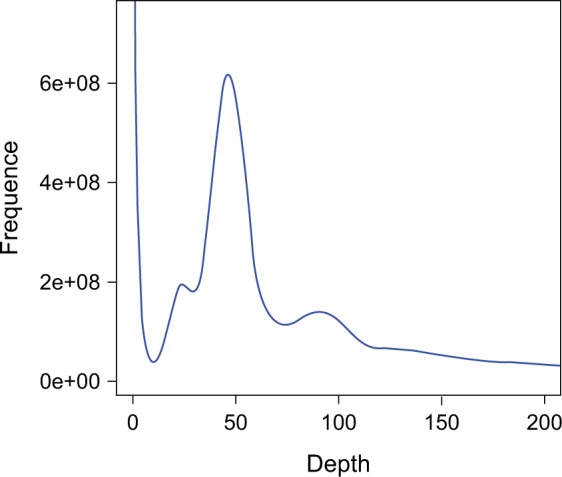
The *k*-mer analysis (*k* = 17) for estimating the genome size of *Nyssa sinensis*. The x-axis refers to the *k*-mer depth; the y-axis refers to the frequency of the *k*-mer for a given depth.

The genome size of *N*. *sinensis* was first estimated using the *k*-mer analysis with Jellyfish^9^. The 17-mer frequency of Illumina short reads followed a Poisson distribution, with the highest peak occurring at a depth of 45 (Fig. 1). The estimated genome size was 1,051.16 Mb, and the heterozygosity rate of the genome was 0.87% (Table 2). In addition, we performed flow cytometry analysis using *Vigna radiata* as the internal standard, and the genome size of *N*. *sinensis* was estimated at 992 Mb.

**Table 2:** Summary of the *k*-mer analysis for estimating the genome size of *Nyssa sinensis*. 150-bp paired-end reads were generated using the Illumina platform, and a total of 58.83 Gb of reads were obtained after adapter trimming and quality filtering. The frequency of each *k*-mer was calculated and plotted in Fig. 1.

*K*-mer	*K*-mer number	*K*-mer depth	Genome size	Heterozygosity rate	Repeat
17	49,922,730,728	45	1,051.16 Mb	0.87%	56.92%

### *De novo* genome assembly and pseudo-chromosome construction

After the self-error correction step, the PacBio long reads were assembled into contigs using the hierarchical genome assembly process (HGAP)^10^ as implemented in the FALCON assembler^11,12^. In addition, two rounds of polishing were applied to the assembled contigs using the Quiver algorithm^10^ with the PacBio long reads, and another round of the genome-wide base-level correction was performed using Pilon^13^ with the Illumina short reads. Finally, the Purge Haplotigs pipeline^14^ was run to produce an improved, deduplicated assembly. The resulting genome assembly of *N*. *sinensis* contained 1,001.42 Mb of sequences in 647 polished contigs with an N50 size of 3.62 Mb (contigs shorter than 100 bp were discarded; Table 3), and the overall GC-content was 35.98%.

**Table 3:** Summary of genome assemblies of *Nyssa sinensis* created at different stages of the assembly process.

	FALCON assembly	Post Quiver	Post Pilon	Post Purge Haplotigs
Size of assembled contigs (bp)	1,060,185,320	1,066,201,220	1,065,961,334	1,001,417,765
No. of contigs (>100 bp)	1,553	1,553	1,553	647
Max. contig length (bp)	29,948,976	30,066,399	30,063,645	30,063,645
Contig N50 size (bp)	3,447,630	3,466,154	3,466,018	3,624,455
Contig N90 size (bp)	525,139	527,948	527,985	1,008,072

**Fig. 2:**
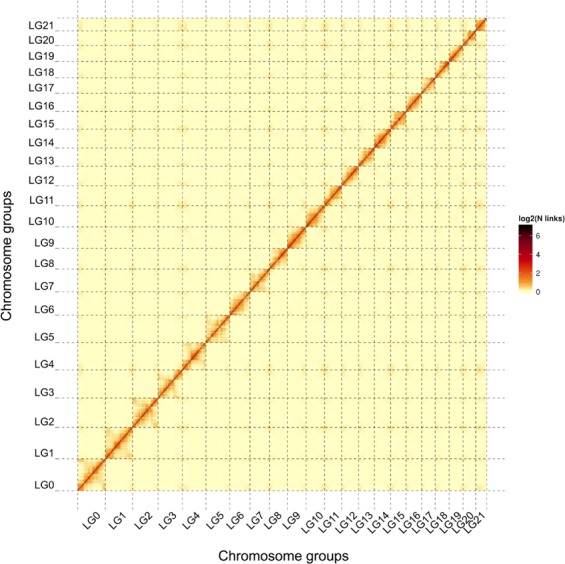
Interaction heat map of Hi-C links among chromosome groups for *Nyssa sinensis*. The assembled genome of *N*. *sinensis* was divided into 100-kb non-overlapping windows (or bins), and valid interaction links of Hi-C data were calculated between each pair of bins. The binary logarithm of each link number is coded using colors ranging from light yellow to dark red, indicating the frequency of Hi-C interaction links from low to high. LG0–LG21 represent the 22 chromosome groups inferred by LACHESIS.

Construction of pseudo-chromosomes followed the previous study^15^ using the Hi-C library. Briefly, the clean Hi-C reads were mapped to the assembled contigs using the Burrows–Wheeler Aligner^16^ (BWA), and only uniquely mapped read pairs were considered for downstream analysis. Duplicate removal, sorting, and quality assessment were performed using HiC-Pro^17^. The assembled contigs were then clustered, ordered, and oriented into pseudo-chromosomes using LACHESIS^18^. A total of 585 contigs spanning 1,000.96 Mb (i.e., 99.95% of the assembly) were clustered into 22 chromosome groups (Fig. 2), matching the chromosome counts in *Nyssa* (*n* = 22) based on cytological studies^19–21^. In addition, of the clustered contigs, 382 contigs spanning 968.49 Mb (i.e., 96.71% of the assembly) were successfully ordered and orientated (Online-only Table 1).

### The annotation of repetitive elements

To annotate repetitive elements in the genome of *N*. *sinensis*, we utilized a combination of evidence-based and *de novo* approaches. The genome assembly was first searched using RepeatMasker (http://www.repeatmasker.org) against the Repbase database^22^. Next, a *de novo* repetitive element library was constructed using RepeatModeler (http://www.repeatmasker.org/RepeatModeler/), which employed results from RECON^23^ and RepeatScout^24^. This *de novo* repetitive element library was then utilized by RepeatMasker to annotate repetitive elements. Results from these two runs of RepeatMasker were merged together. A total of 664.91 Mb of repetitive elements (i.e., 66.40% of the assembly) were identified in the genome of *N*. *sinensis* (Table 4), including retroelements (32.51%), DNA transposons (11.23%), tandem repeats (2.95%), and unclassified elements (19.71%). Thus, the percentage of predicted repetitive elements in the genome of *N*. *sinensis* is much higher in comparison with that in the closely related species *C*. *acuminata* (i.e., 35.6%^5^).

**Table 4:** Summary of repetitive elements annotated in the genome of *Nyssa sinensis*.

Element type	No. of elements	Length occupied (bp)	Percentage of genome (%)
LTR	433,015	284,127,511	28.37
LINE	83,199	40,234,938	4.02
SINE	9,696	1,168,096	0.12
DNA	326,322	112,436,370	11.23
Satellite	2,879	959,079	0.10
Simple repeats	72,249	28,565,346	2.85
Unclassified	582,325	197,421,711	19.71
Total	1,509,685	664,913,051	66.40

**Fig. 3:**
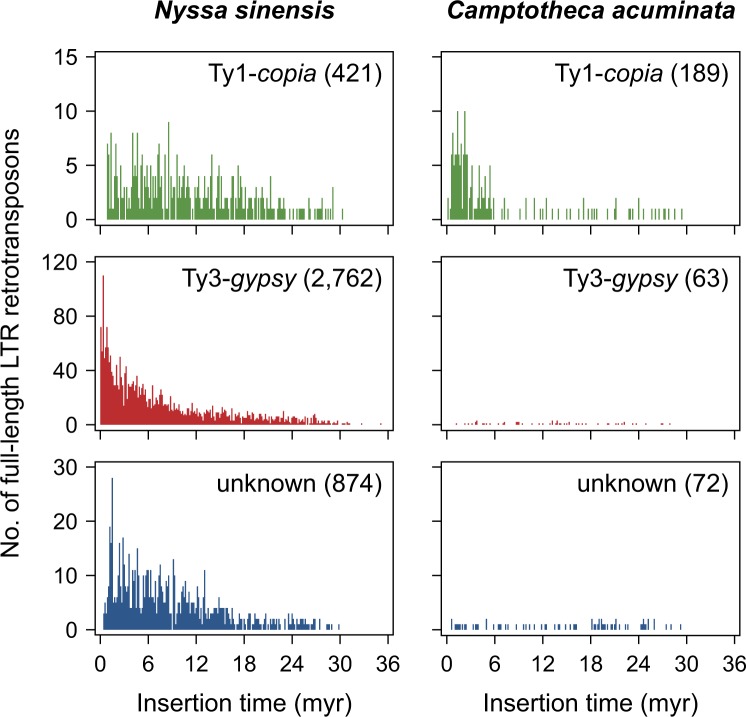
Estimated insertion time of full-length long terminal repeat (LTR) retrotransposons in the genomes of *Nyssa sinensis* and *Camptotheca acuminata*.

Long terminal repeat (LTR) retrotransposons are prevalent in plant genomes^25^. In order to develop high-quality gene annotation, we additionally identified LTR retrotransposons in the genome of *N*. *sinensis* using a combination of four programs (i.e., LTR_FINDER^26^, LTRharvest^27^, LTR_retriever^25^, and RepeatMasker). Here, LTR_FINDER and LTRharvest were used for initial identification of LTR retrotransposons; LTR_retriever was then used to filter out false positives and estimate the insertion time for each intact LTR retrotransposon; finally, RepeatMasker was used for annotation of LTR retrotransposons. Our results suggested that when comparing with *C*. *acuminata*, LTR retrotransposons in the genome of *N*. *sinensis* had recently undergone a rapid proliferation, particularly the Ty3-*gypsy* family (Fig. 3).

### Protein-coding gene prediction and functional annotation

The identification of protein-coding genes in the assembled genome of *N*. *sinensis* was based on transcriptome data and *ab initio* prediction. First, two strategies (i.e., *de novo* and genome-guided assembly) were applied to assemble RNA-Seq reads into transcripts using Trinity^28^. In order to use Trinity in genome-guided mode, RNA-Seq reads were first aligned to the assembled genome of *N*. *sinensis* using HISAT2^29^. These two transcriptome assemblies were then merged. To generate the initial gene models for training AUGUSTUS^30^, our assembled transcripts were processed and utilized to identify open reading frames (ORFs) by the Program to Assemble Spliced Alignments^31^ (PASA). AUGUSTUS was then utilized for *ab initio* gene prediction based on (i) a generalized hidden Markov model (HMM) and (ii) semi-Markov conditional random field (CRF). In addition, extrinsic evidence was incorporated into AUGUSTUS using a hints file, which was generated by aligning RNA-Seq reads to the hard-masked genome assembly with HISAT2. Lastly, untranslated regions (UTRs) and alternative splicing variations were annotated using PASA. A total of 37,884 protein-coding genes were predicted in the genome of *N*. *sinensis* (Table 5).

**Table 5:** Summary of protein-coding genes predicted in the genome of *Nyssa sinensis*.

No. of protein-coding genes	37,884
No. of transcripts	62,426
Average exon size per transcript (bp)	1,949
Average coding sequence (CDS) size per transcript (bp)	1,240
Average intron size per transcript (bp)	7,728
Average exon number per transcript	6.42
Average exon size (bp)	303

For functional annotation, our predicted protein-coding genes were searched against the Swiss-Prot and TrEMBL databases^32^ using BLAST+^33^ with and *E*-value threshold of 1e-05, as well as the InterPro database using InterProScan 5^34^. In addition, for predicted protein-coding genes, gene ontology (GO) annotations were performed using Blast2GO^35^, and KEGG orthology (KO) identifiers were assigned using KEGG Automatic Annotation Server^36^ (KAAS). A total of 36,185 genes (i.e., 95.52% of all predicted protein-coding genes) were successfully annotated by at least one database (Table 6).

**Table 6:** Summary of functional annotation of protein-coding genes in the genome of *Nyssa sinensis*.

Database	No. of annotated genes	Percentage (%)
Swiss-Prot	28,305	74.71
TrEMBL	33,656	88.84
InterPro	35,769	94.42
GO	32,293	85.24
KEGG	8,235	21.74
Annotated	36,185	95.52
Total	37,884	100.00

## Data Records

PacBio Sequel long reads^37^, Illumina paired-end reads^38^, Hi-C reads^39^, and RNA-Seq reads^40^ have been deposited in NCBI Sequence Read Archive (SRA). The genome assembly and annotation of *N*. *sinensis* have been deposited in CoGe^41^, Figshare^42,43^, and GenBank^44^.

## Technical Validation

### Total RNA quality assessment

The quality of total RNA was evaluated using (i) agarose gel electrophoresis for RNA degradation and potential contamination, (ii) NanoDrop spectrophotometer for preliminary quantitation, and (iii) Agilent 2100 Bioanalyzer for RNA integrity and quantitation. Total RNA samples included in this study had an RNA integrity number (RIN) of 9.7–10 and an rRNA ratio of 1.5, which were then enriched for mRNA via an oligo(dT)–magnetic bead method.

### Quality filtering of Illumina data

Illumina raw data were first filtered using Trimmomatic^45^ to remove paired-end reads if either of the reads contained (i) adapter sequences, (ii) more than 10% of N bases, and (iii) more than 20% of bases with a Phred quality score less than 5.

### Assessing the completeness and accuracy of the genome assembly

We first evaluated the completeness of the assembly using CEGMA^46^ and BUSCO^47,48^. Out of the 248 core eukaryotic genes in CEGMA, 235 (94.8%) complete matches and 244 (98.4%) complete plus partial matches were found in the assembled genome of *N*. *sinensis*. In addition, 93.4% complete and 2.2% partial of the 1,440 plant-specific BUSCO genes were identified in the assembly. Second, the accuracy of the assembly was assessed using our Illumina short reads. In total, 94.51% of the filtered short reads (58.83 Gb, Table 1) were mapped to the assembled genome of *N*. *sinensis* using BWA, which covered 99.89% of the assembly. Furthermore, Single-nucleotide polymorphisms (SNPs) were called and filtered using SAMtools^49^, and a total of 5,046,556 SNPs with a sequencing depth between 10× and 100× were identified, consisting of 5,040,788 heterozygous SNPs and 5,768 homozygous SNPs. The low rate of homozygous SNPs (0.0006% of the assembled genome) suggested the high accuracy of the assembly. Finally, the assembled genome of *N*. *sinensis* was divided into 10-kb non-overlapping windows, and the scatter plot of the sequencing depth versus the GC-content based on 10-kb windows indicated no contamination of foreign DNA in the assembly.

## Data Availability

Sequencing data were generated using the software provided by sequencer manufacturers, and processed following the instruction manual of the software cited above. No custom codes were generated for this work.

## Online-only Table

**Online-only Table 1:** Summary of chromosome groups inferred using Hi-C data in the genome of *Nyssa sinensis*.

Chromosome groups	No. of contigs	Size of contigs (bp)
LG0	31	66,977,172
LG1	27	64,355,139
LG2	37	61,373,574
LG3	49	60,749,938
LG4	42	59,935,386
LG5	46	60,314,056
LG6	36	49,697,241
LG7	37	48,508,438
LG8	27	44,304,474
LG9	18	44,257,002
LG10	21	43,776,600
LG11	20	41,982,236
LG12	16	40,386,121
LG13	24	38,768,690
LG14	6	38,017,904
LG15	22	37,846,787
LG16	18	37,775,338
LG17	40	36,742,899
LG18	18	34,721,733
LG19	22	33,452,663
LG20	18	31,025,580
LG21	10	25,997,779
Total clustered (ratio)	585 (90.42%)	1,000,966,750 (99.95%)
Total ordered and oriented (ratio)	382 (59.04%)	968,489,389 (96.71%)
